# Cross-Induction of Anti-Complexing Antibodies in Patients Treated with Botulinum Toxin Formulations Containing Complexing Proteins

**DOI:** 10.3390/toxins18020099

**Published:** 2026-02-14

**Authors:** Yuttana Srinoulprasert, Surachet Sirisuthivoranunt, Chattip Sripatumtong, Tunsuda Tansit, Pornsuk Yamlexnoi, Onjira Meethong, Rungsima Wanitphakdeedecha

**Affiliations:** 1 Department of Immunology, Faculty of Medicine Siriraj Hospital, Mahidol University, Bangkok 10700, Thailand; yuttana.sri@mahidol.ac.th (Y.S.);; 2 Department of Dermatology, Faculty of Medicine Siriraj Hospital, Mahidol University, Bangkok 10700, Thailand; siri.surachet@gmail.com (S.S.); pornsuk.yam@mahidol.ac.th (P.Y.); onjira.mee@mahidol.ac.th (O.M.)

**Keywords:** botulinum toxin type A, complexing proteins, immunogenicity, cross-reactive antibodies

## Abstract

Botulinum toxin type A (BoNT/A) formulations differ in their content of non-toxic accessory proteins, also known as complexing proteins (CPs), which may influence immunogenicity. Some BoNT/A products share structurally similar CPs, potentially leading to antibody cross-reactivity among formulations. This prospective study investigated whether patients treated with different BoNT/A products develop cross-reactive anti-CP antibody responses. One hundred participants were allocated into five treatment groups, each receiving a single BoNT/A formulation: incobotulinumtoxinA (IncoA), onabotulinumtoxinA (OnaA), abobotulinumtoxinA (AboA), letibotulinumtoxinA (LetiA), or prabotulinumtoxinA (PraboA). Each participant received 50 units or equivalent dosing. Serum samples were collected 180 days post-injection, and anti-CP antibodies were quantified using an absorption ELISA and compared with a toxin-naïve control group. IncoA did not induce significant anti-CP antibody responses. In contrast, higher antibody levels were observed in the OnaA, LetiA, and PraboA groups against multiple CPs, suggesting structural similarity and cross-reactivity. AboA primarily induced antibodies directed against its own CPs and those of PraboA. These findings demonstrate that CP-containing formulations can induce cross-reactive antibody responses, whereas CP-free incobotulinumtoxinA exhibits minimal immunogenicity. This study highlights the importance of CP composition in guiding clinical product selection, particularly in patients requiring repeated BoNT/A administration.

## 1. Introduction

Botulinum toxin type A (BoNT/A) injection has established itself as the most frequently performed minimally invasive esthetic procedure worldwide [1]. Beyond aesthetic applications, it serves as a first-line therapy for a broad spectrum of neuromuscular and autonomic disorders, including cervical dystonia, spasticity, and chronic migraine [2,3]. With the rising trend of preventative treatments in younger demographics, patients are increasingly subjected to repeated exposure over decades [4]. The toxin functions by cleaving SNAP-25 to inhibit acetylcholine release, resulting in temporary muscle paralysis [5].

Commercial BoNT/A products consist of a 150 kDa core neurotoxin, with or without additional non-toxic accessory proteins referred to as complexing proteins (CPs), including hemagglutinins and non-hemagglutinating proteins [6]. These proteins naturally surround the neurotoxin in the native bacterial complex and protect it during gastrointestinal passage in natural intoxication; however, their necessity in injectable therapeutic formulations remains debated [6,7].

Immunogenicity represents a key concern in long-term or repeated BoNT/A treatment, particularly the development of antibody responses that may compromise clinical efficacy. While the core neurotoxin itself can elicit immune recognition, CPs have been implicated as potential enhancers of immunogenicity by acting as adjuvant-like proteins [8]. Specifically, hemagglutinin components have been shown to activate Toll-like receptor 2 (TLR2) on dendritic cells, triggering pro-inflammatory signaling [9]. Repeated exposure to CP-containing formulations may therefore increase the likelihood of antibody formation, potentially contributing to secondary treatment failure [6,7,8]. Recent meta-analyses estimate the prevalence of neutralizing antibodies in esthetic indications to range between ~0.2% and ~0.5%, with higher rates observed in therapeutic uses [10].

Currently available BoNT/A products differ markedly in their CP content. OnabotulinumtoxinA, abobotulinumtoxinA, letibotulinumtoxinA, and prabotulinumtoxinA contain varying amounts and compositions of CPs, whereas incobotulinumtoxinA is purified to contain only the core neurotoxin without CPs [7,8,11]. Structural similarities among CPs derived from different formulations may facilitate antibody cross-reactivity, whereby antibodies generated against one formulation recognize CPs from another. Such cross-reactivity could limit the effectiveness of switching between BoNT/A products in patients with suspected immunogenic resistance.

Although anti-CP antibodies have been detected in BoNT/A non-responders using absorption ELISA techniques [12], the extent and pattern of cross-reactivity among CPs from different commercial BoNT/A formulations have not been systematically evaluated in humans. Therefore, this study aimed to investigate whether treatment with specific BoNT/A formulations induces anti-CP antibodies that cross-react with [1] CPs from other formulations, thereby providing clinically relevant insight into formulation-specific immunogenicity.

## 2. Results

### 2.1. Patient Population and Characteristics

A total of 137 participants were enrolled, including five BoNT/A treatment groups (*n* = 20 per group) and a toxin-naïve control group (*n* = 37). All participants were Thai adults aged 18–60 years, with a predominance of female subjects (92.7%). Mean ages were comparable across groups, ranging from 33.45 ± 8.33 to 38.7 ± 8.72 years, with no statistically significant differences. None of the participants exhibited clinical BoNT/A treatment failure during follow-up. Demographic characteristics are summarized in Table 1.

### 2.2. Anti-Complexing Protein Antibody Responses

**Figure 1:**
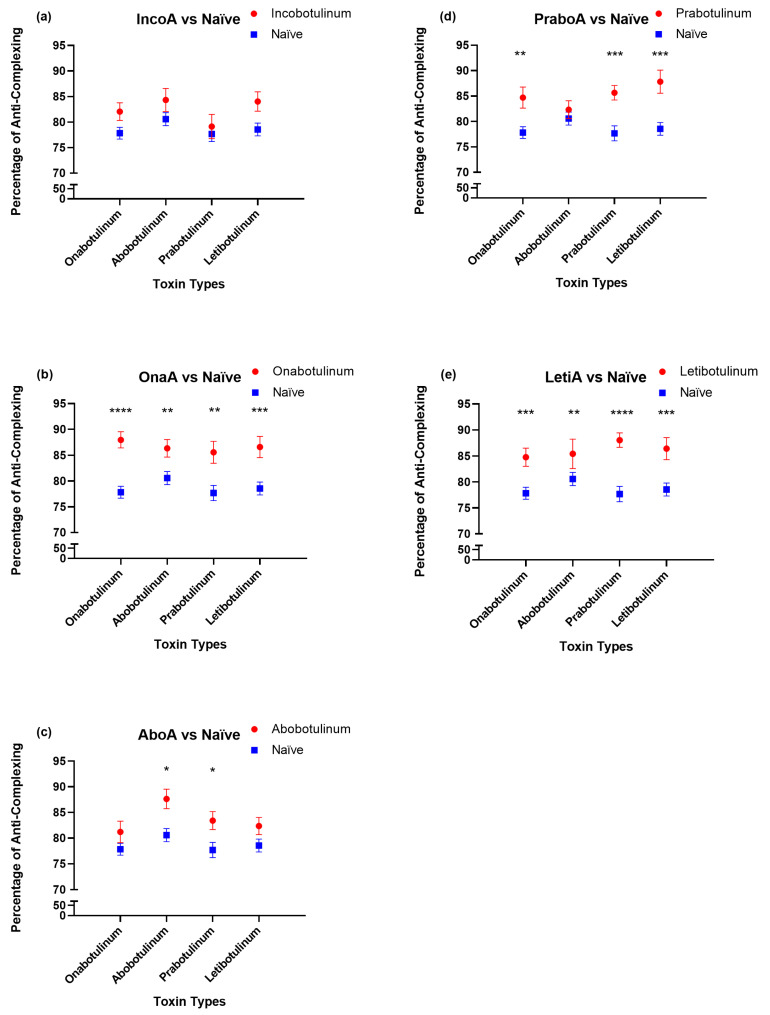
Comparison of anti-complexing protein (anti-CP) antibody levels in sera from participants treated with different botulinum toxin type A (BoNT/A) formulations [(**a**): Treated with Inco BoNT/A, (**b**): Treated with Ona BoNT/A, (**c**): Treated with Abo BoNT/A, (**d**): Treated with Prabo BoNT/A, and (**e**): Treated with Leti BoNT/A] compared with the toxin-naïve group. The levels of anti-CP antibodies were expressed as a percentage of anti-complexing proteins as described in Materials and Methods. The *y*-axis represents the mean percentage of anti-CP antibodies ± SEM, and the *x*-axis indicates the BoNT/A formulation used for ELISA plate coating. Statistical significance between each treatment group and the naïve group is indicated as follows: *p* < 0.05 (*), *p* < 0.01 (**), *p* < 0.001 (***), and *p* < 0.0001 (****).

Anti-CP antibody levels differed markedly among BoNT/A formulations (Figure 1). Sera from participants treated with OnaA and LetiA demonstrated significantly elevated anti-CP antibody levels against all tested BoNT/A formulations compared with the toxin-naïve group (*p* ≤ 0.01 to *p* ≤ 0.0001), indicating broad cross-reactivity. PraboA treatment also induced elevated antibody responses with cross-reactivity against CPs from other formulations, particularly abobotulinumtoxinA and letibotulinumtoxinA.

In contrast, AboA treatment resulted in a more restricted antibody profile, with a modest but significant increase in antibodies primarily directed against its own CPs and those of PraboA (*p* < 0.05). Notably, participants treated with IncoA did not demonstrate any significant increase in anti-CP antibody levels relative to toxin-naïve controls, indicating minimal immunogenicity with respect to CPs (Figure 1).

Collectively, these findings reveal distinct formulation-specific immunogenic profiles, ranging from broad cross-reactive responses to minimal antibody induction, depending on CP composition.

## 3. Discussion

This prospective clinical study provides direct human evidence that BoNT/A formulations containing complexing proteins can induce cross-reactive antibody responses against CPs derived from other commercial products. In contrast, the CP-free formulation incobotulinumtoxinA demonstrated minimal immunogenicity, with antibody levels comparable to those observed in toxin-naïve individuals. These findings extend previous observations regarding CP-associated immunogenicity and provide new insight into cross-formulation immune recognition.

The broad cross-reactivity observed among OnaA, LetiA, and PraboA suggests that these formulations may share structurally similar CP components, which are recognized by the immune system across products [13,14,15]. Manufacturing processes, bacterial strains, and purification techniques are likely to influence CP composition and may account for the observed immunogenic similarities. Conversely, abobotulinumtoxinA exhibited a more limited cross-reactive profile, possibly reflecting differences in molecular weight distribution and CP structure [14]. This distinct immunological signature may have implications for product switching strategies, although further validation is required.

It is important to distinguish between antibodies directed against complexing proteins and neutralizing antibodies against the 150 kDa core neurotoxin. While anti-CP antibodies do not necessarily equate to functional neutralization of BoNT/A activity, their presence reflects increased antigenic exposure and may predispose patients to broader immune recognition with repeated treatments.

Additionally, the detection of heterogeneity levels of anti-CP antibody in individuals may be explained by molecular mimicry. It is well-established that the human immune system is frequently primed by exposure to various *Clostridium* species within the commensal gut microbiota or through environmental contact. Furthermore, standard immunization protocols, such as the tetanus toxoid vaccine, involve proteins that share high structural homology with botulinum neurotoxins [16]. These prior exposures can induce a population of memory B cells and circulating antibodies that recognize conserved, structurally related epitopes on the complexing proteins of BoNT/A. These pre-existing antibodies account for the heterogeneity of basal hIgG levels observed across our study cohorts. To account for this individual variability and to ensure that our data reflected the specific immunogenic impact of the BoNT/A formulations rather than baseline cross-reactivity, we therefore expressed anti-CP IgG levels as a percentage of the individual’s own basal whole-toxin IgG.

Several limitations should be acknowledged. First, this was a single-center study conducted in a homogeneous Thai population, which may limit generalizability. However, this approach minimized confounding factors related to genetic and procedural variability and provides valuable data for an underrepresented population. Second, the sample size was designed to identify immunological patterns rather than to determine the precise incidence of clinical immunoresistance. Finally, although absorption ELISA enables selective assessment of anti-CP antibodies, functional neutralization assays were not performed; therefore, direct correlations with clinical non-response cannot be conclusively established.

From a clinical perspective, understanding formulation-specific immunogenicity is crucial when selecting BoNT/A products for patients requiring repeated treatment. In cases of suspected antibody-mediated treatment failure, immunological testing may inform product choice. When antibody involvement is confirmed or suspected, CP-free formulations such as incobotulinumtoxinA may offer a rational alternative with a lower risk of further immune stimulation [12].

## 4. Conclusions

BoNT/A formulations containing complexing proteins induce cross-reactive antibody responses that may contribute to immunogenic resistance with repeated exposure. IncobotulinumtoxinA, which lacks complexing proteins, exhibits minimal immunogenicity and may be preferentially considered for patients requiring long-term or repeated BoNT/A treatment.

## 5. Materials and Methods

### 5.1. Study Objectives

This study aimed to investigate the presence and cross-reactivity of antibodies against CPs in sera from patients treated with different BoNT/A formulations

### 5.2. Experimental Design and Population

This pilot study was conducted at a single-center, prospective study with five parallel treatment groups and one naïve control group to study antibody responses in humans. One hundred thirty-seven Thai people (males and females) aged 18 to 60 years old were included in the study. They were divided into naïve group (37 people), which are the people who never received botulinum toxin injection before and treatment groups which received the botulinum toxin treatment. Participants were excluded if they had received botulinum toxin injections within 90 days before study initiation, had underlying immunological diseases, or had a documented history of treatment non-response to BoNT/A.

Serum samples used in this study were derived from participants of a previously published study entitled “A Pilot Study of Differences in Antibody Responses of Intradermal and Intramuscular Injections of Botulinum Toxin Type A.” [17]. Written informed consent for serum storage and future use in subsequent research projects was obtained from all participants.

The sample size was appropriate for the exploration objectives of this study. The primary aim was not to establish the precise incidence of immunoresistance to BoNT/A with high statistical power, but rather to identify the existence and characterize the patterns of antibody cross-reactivity across different BoNT/A formulations. This sample size provides sufficient resolution to detect strong, clinically relevant immunological signals and is consistent with sample sizes used in prior foundational studies of immunogenicity [18]. Given the logistical challenges of recruiting patients with specific BoNT/A treatment histories and suspected immunogenic resistance, this sample size represents a practical and ethically sound approach for this initial investigation.

Participants in the treatment arm were evenly assigned into five groups (*n* = 20 per group), with each group receiving a different commercially available BoNT/A formulation: IncoA (Xeomin, Merz Pharma, Frankfurt, Germany), OnaA (Botox, Irvine, CA, USA), AboA (Dysport, Ipsen Ltd, Paris, France), LetiA (Hugel, Hugel, Inc., Chuncheon-si, Gangwon-do, South Korea), or PraboA (Nabota, Daewoong Pharmaceutical Co., Ltd., Seoul, South Korea). All BoNT/A products were obtained from the same lot and batch to ensure consistency throughout the study. Each participant received an injection of 50 units of BoNT/A or an equivalent dose; for AboA, a dose of 125 units was administered in accordance with established conversion ratios [19]. Treatments were performed for esthetic indications, including facial lifting or facial contouring.

### 5.3. Subject Sample Collection and Preparation

Stored serum samples from participants enrolled in the previously published study were used for the present analysis. For the naïve group, serum samples were obtained from baseline blood collections of individuals who had never received botulinum toxin injections. For the treatment group, serum samples collected at 180 days post-BoNT/A injection were analyzed. The 180-day time point was selected because patients commonly receive repeat BoNT/A injections around this interval [20], and this time frame allows for the evaluation of antibody development and persistence.

The study aimed to detect the presence of antibodies against complexing proteins (CPs) of each BoNT/A formulation at 180 days and to assess their potential cross-reactivity with CPs from other products. Blood samples in the original study were centrifuged at 1000 rpm for 10 min at room temperature, and the serum was stored at −20 °C. Prior to analysis, serum samples were transferred to 4 °C one day before use.

### 5.4. ELISA for Detection of Human Anti-Complexing Protein Antibodies (Absorption ELISA)

An absorption enzyme-linked immunosorbent assay (ELISA), developed by our group, was employed to quantify human IgG (hIgG) antibodies specific to the complexing proteins of BoNT/A. Briefly, two 96-well microplates (Corning, NY, USA) were prepared and designated as absorption plates and working plates. Absorption plates were coated overnight (16–18 h) at 4 °C with IncoA (0.028 units/mL; 50 μL/well). Working plates were similarly coated with IncoA (0.028 units/mL), LetiA (0.02 units/mL), AboA (0.116 units/mL), OnaA (0.02 units/mL), or PraboA (0.134 units/mL). Following washing, absorption plates were blocked with 1% bovine serum albumin (BSA, Merck KGaA, Darmstadt, Germany) and incubated at 37 °C for 1 h. Patient sera, diluted 1:50, were added to the absorption plates and incubated at 37 °C for 1 h; this absorption step was performed twice. Absorbed sera, together with freshly diluted unabsorbed sera, were subsequently transferred to BSA-blocked working plates and incubated at 37 °C for 1 h. After washing, horseradish peroxidase–conjugated rabbit anti-human IgG (Agilent Technologies, Santa Clara, CA, USA) was added, followed by tetramethylbenzidine substrate (Dako, Glostrup, Denmark) for color development. The reaction was terminated with 1 N HCl, and absorbance was measured at 450 nm [21]. Sera contain antibodies recognizing both core botulinum toxin and complexing proteins. Absorbed sera without antibodies specific to core botulinum toxin may mostly contain antibodies specific against complexing proteins. The amount of hIgG specific to complexing proteins was calculated by subtracting the levels of hIgG from unabsorbed samples from those of absorbed samples. Due to the heterogeneity of basal levels of hIgG specific to whole BoNT/A (including core and complexing proteins) among individuals, we normalized the amount of hIgG specific to complexing proteins by their corresponding basal levels of hIgG specific to whole BoNT/A, expressing the results as a percentage of anti-complexing protein IgG. The antibodies that were used in the project is shown in Table S3.

### 5.5. Statistical Analysis

Statistical analyses were conducted using GraphPad Prism version 9.5.0. Descriptive statistics were applied to demographic data. Comparisons between toxin-naïve and treated groups were performed using an unpaired *t*-test for parametric data or the Mann–Whitney U test for non-parametric data. A *p* value ≤ 0.05 was considered statistically significant.

## Figures and Tables

**Table 1:** Demographic data of participants from each group.

Group	Female/Male	Mean Age ± SD
IncobotulinumtoxinA group	20/0	38.7 ± 8.72
OnabotulinumtoxinA group	19/1	33.45 ± 8.33
AbobotulinumtoxinA group	17/3	34.55 ± 8.48
PrabotulinumtoxinA group	19/1	37.4 ± 10.03
LetibotulinumtoxinA group	20/0	36.35 ± 6.48
Naïve group	31/6	35.32 ± 6.02

## Data Availability

The original contributions presented in this study are included in the article/Supplementary Material. Further inquiries can be directed to the corresponding author.

## Supplementary Materials

The following supporting information can be downloaded at https://www.mdpi.com/article/10.3390/toxins18020099/s1. Table S1: Mean percentage of complexing protein antibody in sera of naïve group and treatment group; Table S2: Summarize the pharmacological characteristics of the BoNT/A formulations; Table S3: Antibodies used in ELISA [13,22,23,24,25].

## Abbreviations

The following abbreviations are used in this manuscript:

BoNT/A	Botulinum toxin type A
CPs	Complexing proteins
IncoA	IncobotulinumtoxinA
OnaA	OnabotulinumtoxinA
AboA	AbobotulinumtoxinA
LetiA	LetibotulinumtoxinA
PraboA	PrabotulinumtoxinA
