# Supplementary material for: Cross-Induction of Anti-Complexing Antibodies in Patients Treated with Botulinum Toxin Formulations Containing Complexing Proteins
Source: Toxins (Basel). 2026 Feb 14;18(2):99. doi: 10.3390/toxins18020099 (PMC12945223; doi:10.3390/toxins18020099)
Supplement: Supplementary file 1 [file toxins-18-00099-s001.zip › toxins-4102150-supplementary.pdf]

Supplementary Materials: Cross-Induction of Anti-Complexing Antibodies in Patients Treated with Botulinum Toxin Formulations Containing Complexing Proteins

Yuttana Srinoulprasert[1], Surachet Sirisuthivoranunt[2], Chattip Sripatumtong[1], Tunsuda Tansit[1], Thanya Techapichetvanich[2], Woraphong Manuskiatti[2], and Rungsima Wanitphakdeedecha[2],*

Table S1. Mean percentage of complexing protein antibody in sera of naïve group and treatment group

Group of participant	Coating	Average total hIgG against whole BoNT/A (ng/dl)	Treatment group	Naïve group	P-value
IncobotulinumtoxinA	OnaA	1033.07	82.06	77.82	0.080
	AboA	977.24	84.32	80.58	0.171
	PraboA	984.42	79.13	77.66	0.724
	LetiA	930.70	84.04	78.54	0.067
OnabotulinumtoxinA	OnaA	1729.59	87.98	77.82	<0.001
	AboA	1712.68	86.34	80.58	0.002
	PraboA	1592.80	85.58	77.66	0.001
	LetiA	1594.90	86.6	78.54	<0.001
AbobotulinumtoxinA	OnaA	734.45	81.19	77.82	0.131
	AboA	647.16	84.65	80.58	0.011
	PraboA	599.35	83.41	77.66	0.034
	LetiA	674.26	82.35	78.54	0.105
PrabotulinumtoxinA	OnaA	890.36	84.71	77.82	0.001
	AboA	796.35	79.56	80.58	0.107
	PraboA	728.62	82.32	77.66	<0.001
	LetiA	853.74	87.82	78.54	<0.001
LetibotulinumtoxinA	OnaA	890.36	84.78	77.82	<0.001
	AboA	796.35	85.42	80.58	0.005
	PraboA	728.62	88.06	77.66	<0.001
	LetiA	853.74	86.42	78.54	<0.001

Table S2. Summarize the pharmacological characteristics of the BoNT/A formulations [13, 22–25]

	Incobotulinum	Onabotulinum	Abobotulinum	Prabotulinum	Letibotulinum
**Neurotoxin**	Type A	Type A	Type A	Type A	Type A
**Production process**	Chromatog-raphy	Crystallization	Chromatography	anion exchange chromatography	anion exchange chromatography
**Stabiliza-tion process**	Lyophilization	Vacuum dried	Lyophilization	Vacuum dried	Lyophilization
**Molecular weight**	~150 kDa	~900 kDa	~400 - ~500 kDa	~900 kDa	~900 kDa
**Quantity of neurotoxin (ng pro-tein/100 U)**	~0.44 ng/100 U	~0.73 ng/100 U	~0.65 ng/100 U	Not precisely re-ported	Not precisely re-ported
**Ingredient**	Serum albumin, Sucrose	Serum albumin, Sodium chloride	Serum albumin, Lactose	Serum albumin, Sodium chloride	Serum albumin, Sodium chloride

Table S3. Antibodies used in ELISA

Antibody	Host animal	Dilution	Company
Anti-botulinum Neurotoxin Type A	Rabbit	1:250	LSBio (Washington, USA)
Anti-human IgG HRP	Rabbit	1:1000	Agilent Technologies (California, USA)
Anti-rabbit IgG HRP	Swine	1:125	Agilent Technologies (California, USA)
